# The Use and Role of Mesalamine in the Treatment of Ulcerative Colitis: A Literature Review

**DOI:** 10.7759/cureus.89944

**Published:** 2025-08-12

**Authors:** Shalvin Chand, Manvitha Bendagiri Matam, Nehal K Bhatt, Sanathanan Neelakantan Ramaswamy, Samyuktha Harikrishnan, Yashasvi Agarwal, Lubna Mohammed

**Affiliations:** 1 Medicine, Umanand Prasad School of Medicine, University of Fiji, Lautoka, FJI; 2 Research, California Institute of Behavioral Neurosciences & Psychology, Fairfield, USA; 3 Internal Medicine, Gandhi Medical College, Hyderabad, IND; 4 Internal Medicine, PramukhSwami Medical College, Karamsad, IND; 5 Internal Medicine, Government Erode Medical College and Hospital, Erode, IND; 6 Medicine, Gulf Medical University, Ajman, ARE; 7 Internal Medicine, Sir H.N. Reliance Foundation Hospital and Research Centre, Mumbai, IND; 8 Internal Medicine, Jawaharlal Nehru Medical College, Belagavi, IND; 9 Internal Medicine, Dr VRK (Vizarath Rasool Khan) Women's Medical College, Hyderabad, IND

**Keywords:** 5-aminosalicylic (5-asa) agents, inflammatory bowel disease, mesalamine, sulfasalazine, ulcerative colitis

## Abstract

One of the most common forms of Inflammatory bowel disease amongst adolescents, with chronic relapsing and inflammation involving the colonic mucosa, is ulcerative colitis (UC). Though various possible causative theories have been linked to the development of this condition, the standard treatment approach for mild to moderate cases of UC has been mesalamine. Its use in UC has been primarily due to its anti-inflammatory properties, such as the inhibition of inflammatory cytokines, and a less common side effect profile. The main objective of this study has been to highlight underutilized clinical strategies and support evidence-informed decisions regarding the use of mesalamine for both achieving and sustaining remission in patients with UC across varying age groups. Hence, through this paper, the key results and findings, such as the modest risk of repercussions associated with mesalamine, its suitability for prolonged use, and its chemoprotective feature, will provide more confidence among both physicians and patients in using mesalamine for UC.

## Introduction and background

Ulcerative colitis (UC) is a chronic, disabling, autoimmune condition, with inflammation of the bowel that begins in early adulthood and lasts throughout life [1]. It is most common among the younger population with peak age onset between 15- to 25-year-olds, with equal incidence in both genders [2]. The overall estimated rates of both incidence and prevalence for UC span from 3-15:100,000 and 50-80:100,000, respectively, and even greater numbers are observed in technologically advanced countries [2]. Hence, its global burden is prominent across the most productive age group in the general population, which is a risk for low productivity outcomes globally. Its natural course involves frequently relapsing inflammation affecting the mucosal layer of the colon [3]. The disease usually starts with the rectum, with about 40-50% of patients having the disease confined to the rectum and the rectosigmoid colon area [3]. However, it may also involve and extend proximally to affect the other parts of the colon, or the entire colon could be diseased, with statistics of about 30-40% patients having UC extension beyond the sigmoid flexure and about 20% having the involvement of the entire colon (pancolitis) [3]. 

Diet is one of the most common risk factors in both the development and the clinical progression of IBD, such as UC, though the exact correlation is poorly understood at present [4]. The ever-popular Western diet, with meals rich in proteins, fats, and oil contents and low fruit, vegetables, and fiber consumption, has been linked to the potential reasons for the increasingly alarming rate of IBD [5]. UC with its debilitating symptoms like joint pains, frequent rectal bleeding, weight loss, and frequent bowel movements significantly impacts the socioeconomic and employment quality indicators [1]. It is also linked to increased susceptibility to developing colorectal cancer [1]. 

5-Aminosalicylic acid (5-ASA) agents like mesalamine use are getting increasingly popular among patients with UC [5]. Data from two meta-analyses of induction studies have reported a mean remission rate of 42%, compared to placebo-treated patients [3]. The other two meta-analyses showed that about 58.7% to 72% of patients with UC had no remission [3]. While mesalamine use in clinical practice for UC is the norm, many patients are treated aggressively with steroid-based therapies, which are effective but do come with adverse effects in most of them, exploring the role of effective use of 5-ASA agents like mesalamine will give an alternative to steroids in managing mild to moderate cases of UC with potentially better safety among these patients [3]. Hence, we believe that overreliance on steroids could be minimized, and the oral formulation may enhance the probability of inducing clinical remission in UC and potentially give the practicing physician an alternative, for a steroid-free medicine for patients with mild to moderate UC [3]. In contrast, while mesalamine is considered a safe drug, rare and serious renal toxicity is a possibility, and higher oral pill consumption may raise some compliance issues [6]. Systemic corticosteroids are commonly relied upon as the first step in care for IBD patients who have critical forms of the disease, or among those not responding to amino salicylates such as mesalamine [7]. In clinical trials, the frequently utilized tool to examine endoscopic evidence of the disease in UC is the Mayo endoscopic score (MES). Presently, endoscopically confirmed remission is represented with an MES score of 0 and 1, with vast studies showing that MES of 1 is linked to greater rates of relapse and MES of 0 is the preferred optimal target, with a need to optimize the dose of 5-ASA such as mesalamine to reduce relapse [8]. While mesalamine has been used for decades as the first line of treatment for IBDs, its chemopreventive ability against colorectal cancer is something unique to it [9]. Its use has demonstrated both efficacy and safety in managing UC [10]. Since there are various pathways and cytokines involved in UC pathology, it would be reasonable to hypothesize that many combinations of pharmaceutical agents may aid patients in responding to treatment [11]. Hence, through this literature review article, we aim to discuss the essential role and the clinical use of mesalamine, along with other options available in controlling symptoms, and the remission of the disease amongst patients with UC. 

## Review

Search strategy

Inclusion and Exclusion Criteria

In this traditional review, free full-text articles published in English from the last 10 years that included periods between January 2015 to June 2025, based on human studies, were included. The databases used were PubMed and PubMed Advanced Search. Regular search terms "mesalamine " and "ulcerative colitis " were used as search strategies. The included articles contained information regarding participants, both young (child) and adults of both male and female sex, who were trialed, prescribed, or studied for the use of mesalamine for UC. All preprints published papers more than 10 years ago, in foreign languages, based on animal studies, UC managed with other monotherapy, or participants not on mesalamine, were excluded to avoid bias and seek pertinent information needed to study the role of mesalamine in UC. Moreover, any conference abstracts, posters, or presentations that lacked full-text articles were excluded due to limited data availability.

Discussion 

This section will discuss the approach of using 5-ASA agents as the first-line medication for managing UC. Moreover, we will discuss the etiology and pathophysiology of UC, its clinical presentation, diagnosis, and the current standard therapy options for UC treatment, along with the 5-ASA agents. A more in-depth review of various studies demonstrating the use and role of 5-ASA (mesalamine) has also been summarized, along with the latest advances in UC management. 

*Etiology and Pathophysiology of UC* 

While UC remains identified as an idiopathic condition, various possible causes of UC have been assumed and include infections, dietary components, impaired immune response to bacteria or one’s self-antigens, along with possible environmental causes [1]. However, it seems more likely that the normal intestinal microflora may be a key determinant in the emergence of IBD, such as UC [1]. Interestingly, in the same study, smoking had demonstrated a protective effect in UC compared to Crohn’s disease, with a high risk of UC occurrence post-two years smoking cessation [1]. 

*Diet* 

In terms of diet, the prospective study involving over 400 patients by Barnes and colleagues explored the relation between UC flares and dietary intake of certain fatty acid foods [5]. These flares could be due to certain changes to the gut microbiome, exposure to direct dietary antigens, or changes in gut permeability after certain food consumption [5]. It also revealed that high intake of myristic acid (found in palm oil derivatives, dairy fats, and coconut oil) was linked to high odds of relapse [5]. This study also found that while previous studies had linked processed meat, alcohol, and sulfur products to UC flares, they did not find any strong evidence of such, yet acknowledged the fact that larger and further studies are needed to investigate the relationship between diet and UC flares. 

Generally, in terms of pathophysiology, a state of impaired immune response leads to sustained mucosal inflammatory activation with resultant recruitment of inflammatory mediators such as leukocytes from the gut vessels, leading to the mucosa being primarily composed of non-T helper lymphocyte cell population, CD4+ cells, triggering a humoral immune response [1]. Moreover, this uncontrolled immune activation, coupled with improper suppression, is the main mechanism in UC [1]. Furthermore, the chronic inflammation in UC demonstrates high levels of proinflammatory signaling molecules such as IL-1, IL-6, IL-8, and TNF-α that result in continued tissue damage and immune dysfunction [1]. Hence, the need to study the role of mesalamine in controlling this autoimmune overdrive in this literature review. 

Clinical Presentation 

Patients with UC typically present with varying degrees of severity of the symptoms, depending on the extent of the disease involved, and a symptom-free period between flare-ups [1]. The typical trial is a relapsing-remitting course, with around 10% of cases exhibiting acute fulminating episodes [1]. Due to its inflammatory involvement of the colonic mucosa, the most common presentation is blood in stool and diarrhea [12]. While the actual clinical picture depends on the disease extent, patients with left-sided colitis or proctitis generally complain of urgency and incomplete fecal evacuation, a small percentage, around 10% with constipation, in contrast to patients with pancolitis, who have abdominal pain and bloody loose stools [12]. On physical examination, signs of anemia, blood on per rectal exam, and abdominal tenderness may be present; moreover, anal fissure and tags result due to chronic diarrhea, though it is more common in Crohn's disease. Furthermore, Clostridium difficile must be ruled out during workups since it carries a high risk for surgeries and mortality, as well as being a notorious precipitant leading to flare-ups [12]. Hence, in general, the common symptoms of UC include episodic crampy abdominal pain, sporadic rectal bleeding with mucus, straining to defecate, and mild loose stools (diarrhea) [1]. In more severe cases, patients experience weight loss, anemia, fever, and malnutrition [1].

In terms of extraintestinal presentations, about one-third of UC patients develop these manifestations, and up to a quarter of patients show signs of these manifestations even before being diagnosed with inflammatory bowel disease [12]. Peripheral arthritis is the most common extraintestinal manifestation, along with pyoderma gangrenosum and primary sclerosing cholangitis, being more prevalent among UC than the Crohn’s disease population [12]. Moreover, the risk of venous thromboembolism increases up to three to four times among patients with inflammatory bowel disease, especially during flare-ups and hospitalization, where corticosteroid therapies are needed [12]. Hence, there is a need to ensure prophylaxis against venous thromboembolism in these patients.

*Diagnosis* 

The overall analysis of clinical presentation, laboratory results, endoscopic, histologic, and radiological findings is the hallmark of diagnosing UC, rather than by any single study [13]. Disease severity classification and disease progression are predicted with more invasive procedures, such as Endoscopic findings [1]. Patients with a clinical presentation listed above, endoscopic findings of friable, ulcerated, and edematous mucosa, along with biopsy showing inflammatory infiltration, crypt abscesses, and widespread superficial ulceration, suggest UC [1].

Generally, anyone with a suspected possibility of UC must have stool examinations, specifically stool cultures and Clostridium difficile studies, to rule out any colonic infections [12]. Patients can present with low hemoglobin levels, elevated white cells and thrombocytosis, but low albumin levels are indicative of severe disease and a strong predictor of treatment failure with biologic medications and need for surgical intervention such as colectomy [12].

Other inflammatory markers, such as C-reactive protein and erythrocyte sedimentation rate (ESR), may be elevated [12]. Also, non-specific perinuclear antineutrophil cytoplasmic antibodies may be raised in UC [12]. Ultimately, endoscopic examination with multiple biopsies is the best way to establish and confirm the diagnosis of UC [12]. 

*Biomarkers in UC* 

It is understood that evaluating the colonic wall histological activity may help guide treatment options and indicate the prognosis of UC [14]. Moreover, patients in histological remission are presumably symptom-free and demonstrate decreased risk of relapses and can avoid hospital visits, surgery, and reduced chances of developing colon cancer [14]. Nonetheless, biopsy and invasive procedures like endoscopy are not patient-friendly [14]. Fecal calprotectin is a non-invasive biomarker that relates well to the colonic inflammatory histological activity, hence a great tool for colonic histological assessment in UC [14].

Calprotectin is identified as a key teeming protein in the neutrophils that is released into the colon during inflammation [15]. It is a dimer formed by the association of S100A8 and S100A9 proteins, which chelate iron and Zinc, conferring antibacterial action. Due to its greater stability, in feces, it is easily a good choice for a non-invasive biomarker and is considered superior to the FIT (fecal immunochemical) test in terms of its diagnostic value [15]. Additionally, studies have shown that patients in clinical remission had fecal calprotectin levels of less than 50mg/l with normal colonoscopy findings. Thus, this highlighted the use of fecal calprotectin in assessing mucosal healing in IBD patients [16]. Several recent studies have compared the C-reactive protein levels in both CD and UC, respectively, and have demonstrated that the concentration levels of CRP decreased in those treated with infliximab; this result makes CRP an effective tool in both treatment efficacy and monitoring flare-ups [17]. Hence, both fecal calprotectin and CRP levels guide treatment by indicating the severity of the inflammation or active vs clinical remission status of UC.

 *Genetic Testing* 

In the pediatric population, the concept of genetic testing is gaining momentum. It is believed that approximately (lower than 0.5%) pediatric subsets of individuals with IBD have a monogenic form of the disease [18]. The National Health Service test (Genomic Medicine Service R15 panel) has commissioned the screening of approximately 70 genes linked to monogenic IBD and is available to children with onset before two years of age [18]. This may provide earlier diagnosis and help streamline the treatment and prevention approach towards patients at risk of developing UC.

*Treatment and Management of UC with Mesalamine* 

**Figure 1:**
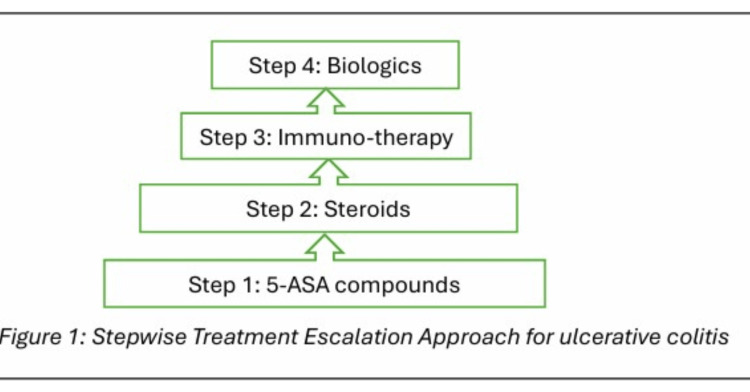
Stepwise Treatment Escalation Approach for Ulcerative Colitis 5-ASA: 5-Aminosalicylic acid Adapted from ref [19] under Creative Commons Attribution-NonCommercial 4.0 International License

Sulfasalazine, an oral 5-ASA compound, was first developed for UC. Following ingestion, colonic microbiomes split this compound into its active (mesalamine) and inactive (sulfapyridine) forms, respectively [19]. Several other pro-drugs include balsalazide, olsalazine, and sulfasalazine; these are reduced to 5-ASA by azoreductase present in the gut microbiome [20]. Mesalamine is the 5-ASA-based agent currently indicated for UC [1]. This active portion of sulfasalazine is devoid of the transport molecule sulfapyridine, which is the primary cause of the side effects [1]. Hence, due to this property of not having the sulfapyridine molecule, mesalamine is preferred over sulfasalazine. It is often given directly or as a prodrug, Olsalazine, to both induce and maintain remission in UC [1]. Other forms of preparation, such as enemas or suppositories, are preferred for mild to moderate distal UC. At the same time, combination oral and topical mesalamine is recommended for colitis on the left side [19]. The flow chart in Figure 1 shows the stepwise treatment escalation approach towards UC management [19].

A technical review paper conducted by the American Gastroenterological Association (AGA) on clinical guidelines for UC discussed the need to optimize the dose of mesalamine for patients with mild to moderate UC before considering any immunosuppressive options [6]. Their research highlighted that combining both oral and suppository mesalamine may be more effective than standard-dose oral 5-ASA alone, among patients with extensive UC or those with symptoms not well controlled with oral medication [6]. The study also indicated that this combination option led to much quicker onset of relief, was welcomed by patients, and may have been due to a larger effective dose and the synergistic effect of the topical anti-inflammatory benefits of suppository mesalamine [6]. While this combination therapy might be helpful for patients on maintenance therapy trying to avoid immunosuppressive medications, compliance with suppository therapy is generally low and only preferred during episodes of flare-ups [6]. Another study, conducted by Fukuda and colleagues, aimed to evaluate the effectiveness of oral 5-ASA dose escalation in UC patients with a Mayo endoscopic subscore (MES) score of 1 [8]. Their study concluded that increasing the dose of 5-ASA in UC patients lowered their risk of relapse over one year; these benefits were evident in patients using immunomodulators. Other findings suggested that steroid use was linked to greater relapse risk, though more research has been recommended [8]. In terms of the use of probiotics, a review published by the British Journal of Pharmacology analyzed various systematic reviews and meta-analyses, which yielded mixed results on the effectiveness of probiotics in UC and concluded that it played a limited role in UC management [7]. Furthermore, another study published in 2024 examined the potential beneficial effects of mesalamine on intestinal epithelial cells in humans [21]. It revealed that mesalamine represses mi-155 expression provoked by inflammation and raises the MMR protein levels, implying a role in cancer prevention for patients with primary sclerosing cholangitis (PSC) and simultaneous UC [21]. Patients with concurrent PSC and UC have a greater risk for colon cancer, perhaps due to extended bile acid exposure resulting in genomic volatility and resistance to programmed cell death. Therefore, the study confirmed that miR-155 increased expression in cancer models triggered by inflammation and illustrated its link to decreased MMR protein levels in lipopolysaccharide-treated cells [21]. Hence, these findings demonstrate mesalamine’s potential as a chemoprotective medication.

*Future Modalities* 

In general, the quality of life is severely impaired due to the incurable and recurrent nature of the disease process in UC [22]. The use of anti-TNF therapies such as Infliximab and adalimumab is remarkably changing the approach from just symptomatic remission to more sustained remission [23]. Moreover, the alternating feature between active and inactive phases of UC among patients has shown high expressions of hub genes, due to damage to the intestinal mucosa caused by pyroptosis in intestinal epithelial cells and raised pro-inflammatory factors, which are believed to be the reason for recurrent disease [22]. Thus, for future treatment, areas of research need to be focused on pyroptosis development, which may help avoid recurrence [22]. Furthermore, a KEGG (Kyoto Encyclopedia of Genes and Genomes) analysis revealed that various pathways like the NOD-like receptor, NF-Kappa B signaling, TNF, and IL-17 signaling are involved in UC and serve as a key area for new drug development and disease treatment [24].

Another growing area of interest regarding the management of patients with UC is the fecal microbiota transplantation (FMT) [25]. As the composition of gut microbiota is different among patients with UC compared to healthy populations, the growth of opportunistic pathogenic bacteria and the decreased presence of symbiotic or gut-friendly bacteria are the main characteristics in an IBD patient [25]. In FMT, feces from a healthy donor are selected and prepared into dehydrated compounds and irrigated into the gastrointestinal tracts of patients. Not only does this procedure restore the diversity of gut bacteria, but it also eliminates the unwanted pathogenic microbes and their pathogenic factors in the colonic environment [25]. Some studies support these, which highlighted that C. difficile and its virulent gene cdtB were eradicated and undetected post FMT procedure with follow-ups at three- and six-month intervals [25]. Furthermore, it was found that in patients with Clostridioides difficile infections, the pre- and post-FMT levels of pathogenic bacteria and their virulent genes both decreased and became undetectable after the FMT procedure [25]. These are some of the potential areas of further research and clinical trial advancements that are and could further help with UC management.

*Mesalamine Review* 

Generally, mesalamine comes as oral delayed-release formulations with about 25 hours of half-life [10]. Patients of varying age groups have been reported to be able to tolerate mesalamine well with only minor adverse and dose-related side effects [26]. Common adverse effects of mesalamine reported are headache, rash with pruritus, feeling nauseated, dyspepsia, and vomiting [27]. Hence, it may be a good suggestion to take the medication with meals and plenty of fluid, which may help with both patient adherence and infrequent side effects. Moreover, severe adverse effects but rare ones include inflammation of the pancreas, pericarditis, pneumonitis, and toxicity to the hepatocellular tissue [27].

Table 1 provides a summary of different trials with the use of mesalamine amongst UC patients highlighting different combination therapies and findings.

**Table 1:** A Summary of Different Trials with the Use of Mesalamine Among UC Patients Highlighting Different Combination Therapies and Findings UC: Ulcerative colitis

Study No.	Author	Publication year	Study type	Intervention/Objective	Findings
1	Tang-Fichaux et al [9].	2021	Review article	To find the link between intestinal inflammation and PKS+ E. coli in colorectal CA occurrence. Also, to find out if mesalamine had any role in Colorectal CA prevention due to Colibactin-producing bacteria.	Findings suggested that mesalamine had two vital properties in terms of chemoprotection. One being able to decrease the severity of mucosal inflammation and the other being able to block the carcinogenic activity of PKS+ E. coli bacteria [9].
2	Krzyzak et al. [10].	2018	Case report review article	This case report evaluated a 38-year-old female patient with an acute flare of ulcerative colitis, on both prednisolone and 5-ASA, with the incident of sinus bradycardia [10].	Findings were that within 24 hours of starting 5-ASA, the patient developed sinus bradycardia, and the heart rate normalized in 5 days post-withholding of 5-ASA. More such analyses and studies were recommended [10].
3	Ye et al. [14]	2021	A Meta Analysis	This study was carried out to evaluate the diagnostic value of fecal calprotectin (FC) in detecting histological inflammation in ulcerative colitis (UC)	Fecal calprotectin (FC) showed strong potential as a noninvasive biomarker for assessing histological response and remission in ulcerative colitis (UC). Moreover, a cut-off range of 100–200 µg/g may help identify patients with mucosal healing and decrease the need for more invasive approaches such as endoscopy and biopsy. However, a more precise threshold value should be tailored to take into consideration variability and disease prevalence, and further studies are encouraged to confirm its clinical use.
4	Chu et al. [28]	2023	Meta Analysis	RCT studies were analyzed in this meta-analysis, comparing the biologic therapies used for UC in both induction and maintenance therapy	The study found that both infliximab and vedolizumab had greater clinical efficacy in induction and maintenance (vs mesalamine). Also, Ustekinumab was found to have fewer adverse effects compared to other biologics used in UC [28].
5	Kaur et al. [29]	2020	Systemic review	In this study, a total of 14 studies (865 randomized participants) were reviewed in both adult (12 studies) and pediatric (two studies), comparing the use of probiotics to mesalamine, probiotics to placebo and combination of probiotic/5-ASA compared to 5-ASA on its own, for inducing remission in UC cases.	This study provided very low yield evidence that probiotics may lead to clinical remission in UC when compared to placebo and probiotic vs 5-ASA. It also highlighted the need for more future research on this topic [29].
6	Jhakri et al. [30]	2025	Systemic review	This study was to evaluate the occurrence of myocarditis being linked to the use of 5-ASA vs the extraintestinal cause of myocarditis among patients with IBD. Papers from the years 2014 to 2024 were analyzed, which included 43 patients.	Out of the 43 patients analyzed, 29 (67%) developed myocarditis which was linked to 5-ASA, while 14 had myocarditis unrelated to 5-ASA medication. This study also highlighted that 5-ASA agents like mesalamine could be causing myocarditis due to mechanisms such as direct cardiac muscle toxicity, Ig E related allergy, and cell-mediated hypersensitivity. While patients did seem to improve following stopping medication and immunosuppression therapy, more research is suggested to differentiate drug-induced from extra-intestinal myocarditis in IBD cases [30].
7	Barberio et al. [31]	2021	Systematic Review and Network Meta-Analysis	This study aimed to evaluate and compare the efficacy of different therapeutic strategies involving 5-aminosalicylates (5-ASAs). It involved analysis of the optimal formulation, dosage, and administration route for its use in ulcerative colitis	The study's findings align with the existing evidence that higher doses of oral mesalamine are more effective for inducing remission in ulcerative colitis compared to combination therapy. In addition, it shows significantly greater efficacy than lower doses. Future randomized controlled trials are recommended to assess the role of combination therapy in the induction of remission and to determine the optimal dosing strategies for oral 5-aminosalicylic acids (5-ASAs) in preventing relapses.
8	Jairath et al. [32]	2015	Systemic review	This review aimed to perform a meta-analysis of randomized controlled trials (RCTs) to quantify placebo response and remission rates in ulcerative colitis, and to examine how these rates have evolved. Moreover, a meta-regression analysis was conducted to identify trial design patterns that influence the placebo effect.	The study revealed that several design factors, such as baseline endoscopic severity, rectal bleed scores, treatment class, disease duration, and timing of outcome measurements, influenced the placebo response and remission rates seen in ulcerative colitis. Furthermore, patient-reported or subjective outcomes showed greater placebo effect, highlighting the importance of endoscopy as a form of objective assessment. Hence, these findings could potentially reduce the participant requirements in ulcerative colitis trials.
9	Awadhi et al. [33]	2023	Consensus review	This review was a Middle East expert consensus meeting, held to develop practical, standardized guidance for clinicians managing UC in this region. They discussed the factors such as medication type, dosage, delivery method, treatment duration, and safety considerations to effectively manage mild to moderate ulcerative colitis	This landmark Middle East expert consensus presented eight Delphi-based recommendations for the management of mild to moderate ulcerative colitis. The guideline aimed at standardizing care, enhancing treatment outcomes, and improving patients’ quality of life by addressing therapy induction, maintenance, and adjustment strategies. Implementation must be tailored to each patient’s clinical profile. Some key elements of the review were that Once-daily dosing of oral 5-aminosalicylates enhances treatment adherence in ulcerative colitis patients (unanimous agreement: 16/16). If patients with mild to moderate ulcerative colitis show no clinical improvement within 4 weeks, treatment escalation is advised (93.7% expert consensus). Additionally, maintaining oral 5-aminosalicylates at doses ≥ 2 g/day is strongly recommended to sustain remission and reduce colorectal cancer risk (100% agreement). Furthermore, Budesonide at 9 mg/day is recommended for eight weeks as an adjunct therapy in mild to moderate ulcerative colitis patients who are unresponsive or lose response to an optimized dose of 5-aminosalicylates, with full expert consensus (100%).In addition, expert consensus supported using optimized 5-ASA dosing, budesonide MMX for nonresponse, and re-escalating therapy if control is lost. Moreover, High-dose 5-aminosalicylates (≥4 g/day) are not linked to a greater risk of adverse events or kidney-related complications compared to lower doses (≤2 g/day), with full expert agreement (100%). For mild to moderate ulcerative colitis, an increase in oral 5-aminosalicylates to ≥4 g/day if patients don’t respond to <4 g/day or if symptoms recur is advised. Full expert agreement (16/16). Finally, combine oral 5-ASA (2.0–4.8 g/day) with topical 5-ASA (either suppository or enema ≥1 g/day) for 2months to help induce remission in mild to moderate proctitis or left-sided UC/pancolitis, with full expert agreement (16/16).
10	D’Amico et al. [34].	2024	A Global Survey	An international survey was carried out to explore how patients with mild-to-moderate ulcerative colitis are managed and treated, with specific attention to optimizing therapy and ensuring consistent follow-up.	This survey provided an overview of how mild-to-moderate ulcerative colitis is managed across different regions of the world. Findings revealed that oral plus rectal 5-ASA remained the preferred initial therapy, while budesonide MMX and systemic corticosteroids are options when 5-ASA proves ineffective. Moreover, although the therapeutic goal is shifting toward achieving deeper remission, endoscopy continues to be the primary guide in everyday clinical decision-making. The growing use of non-invasive tools like biomarkers and intestinal ultrasound (IUS) is promising, yet physicians still express concerns about their clinical utility in practice.

*Limitations* 

Some of the factors that may have limited our interpretation of this literature review are restricting our search to free full-text and English-language articles only, which may create an access and language bias, leading to missed relevant data from non-English papers or geographic regions. Secondly, the clinical severity of UC, the time lag in diagnosis and symptoms onset have not been studied in-depth in the articles researched, hence potentially limiting the ability to generalize the use of mesalamine for UC in our review article. Thirdly, the scope of studies reviewed did not specify any specific patient population, which may have had a varying risk profile or treatment response to mesalamine. 

## Conclusions

The relapsing and chronic nature of UC, with underlying inflammatory activity in the colonic mucosa, requires the need for utilization of 5-ASA compounds like mesalamine to both induce and maintain remission in mild to moderate cases of UC. The efficacy and administration of mesalamine as delayed-release oral pills have been proven to be both safe and have minimal rare adverse effects, such as renal toxicity, for which periodic renal function test is recommended. In addition to its ability to reduce inflammatory cytokines such as TNF-α, IL-1, IL-6, and IL-8 in the colonic mucosa, it was also found that mesalamine use had chemoprotective activity as well. It could also be a perfect steroid-sparing option among patients who would benefit from this steroid-free medicine. Therefore, the purpose of this paper has been to highlight and create more awareness among both physicians and the public regarding the utilization of mesalamine for UC. Furthermore, the chemoprotective action of mesalamine against colonic carcinoma development needs to be researched further, possibly with more case-control trials amongst human participants. This kind of further study may perhaps help create a more pharmacologically viable option, considering the relatively low side effect profile of mesalamine.
